# Relationship between DMFT index and number of pregnancies: a cross-sectional study on enrollment phase of the Tabari Cohort Study

**DOI:** 10.1186/s12903-021-02004-1

**Published:** 2021-12-15

**Authors:** Nadia Elyassi Gorji, Pegah Nasiri, Ali Malekzadeh Shafaroudi, Zohreh Shahhosseini, Zeinab Hamzehgardeshi, Mahmood Moosazadeh

**Affiliations:** 1 Dentistry Student, Student Research Committee, Faculty of Dentistry, Mazandaran University of Medical Sciences, Sari, Iran; 2 Sexual and Reproductive Health Research Center, Mazandaran University of Medical Sciences, Sari, Iran; 3 Department of Reproductive Health and Midwifery, Sexual and Reproductive Health Research Center, School of Nursing and Midwifery, Mazandaran University of Medical Sciences, Sari, Iran; 4 Gastrointestinal Cancer Research Center, Non-Communicable Diseases Institute, Mazandaran University of Medical Sciences, Sari, Iran

**Keywords:** Number of pregnancies, DMFT index, Tooth decay

## Abstract

**Background:**

People of all age and gender groups are at risk of dental diseases; however, some groups, such as pregnant women, are more vulnerable than others due to their specific physiological situations. The protection of maternal and fetal is critical. Therefore, the present study intended to investigate the relationship between the Decayed, Missing, and Filled Teeth (DMFT) index and the number of pregnancies.

**Methods:**

The present cross-sectional study was performed using the Tabari Cohort Study (T.C.S.) data. Data of 5,496 women enrolled in the T.C.S. were included in the study. After obtaining the approval of the Ethics Committee, the related data on the variables of age, age at the first pregnancy, number of pregnancies, total number of teeth at the time of examination, number of DMFT, employment status, socioeconomic status, educational level, residence location, body mass index, and the status of diabetes mellitus, hypertension, and cardiovascular diseases were extracted. Finally, data analysis was performed in the STATA software (version 14).

**Results:**

The mean DMFT index in women with 1, 2, 3, 4, 5, 6, and more than six pregnancies were obtained at 12.74 ± 7.11, 13.09 ± 7.06, 14.80 ± 7.81, 17.07 ± 8.11, 19.82 ± 9.02, 22.89 ± 8.98, and 26.17 ± 8.01, respectively (*P* < 0.001). Using the multivariate linear regression and adjusting the effect of potential confounding variables, it was found that the DMFT index increased by 34% for each unit increase in the number of pregnancies (β = 0.34, *P* < 0.001).

**Conclusions:**

According to our results, there was a significant relationship between the DMFT index and the number of pregnancies. The DMFT index was increased with an increased number of pregnancies. Therefore, oral healthcare promotion should receive special attention in healthcare planning and related policies by raising awareness and providing easy access to dental services for women of childbearing age, especially pregnant women.

## Background

Dental health plays an essential role in general health; therefore, poor oral health and untreated or dental diseases can adversely impact the quality of life [1]. People of all age and gender groups are at risk of dental diseases; however, some groups, such as pregnant women, are more vulnerable than others due to their specific physiological situations. The protection of maternal and fetal health is greatly important. Pregnant women are prone to gingival diseases and tooth decay due to the specific hormonal and nutritional changes during pregnancy.

Moreover, they may have problems with routine healthcare measures that are easily taken for non-pregnant women [1]. It has been shown that 58–65% of pregnant women lack a favorable dental condition [2]. On the other hand, dental caries can lead to adverse consequences since the pain experienced and subsequent stresses negatively affect the quality of life of pregnant women [3]. Furthermore, the pain may make them take painkillers or other medications with unpredictable effects on the fetus [4].

The increased prevalence of dental diseases in pregnant women can be due to poor oral hygiene. Pregnant females suffer from nausea, vomiting, and gastroesophageal reflux [5], which can inhibit them from maintaining appropriate oral health. Additionally, carbohydrate intake increases up to 63% in pregnant women due to improper diet [6], which increases the risk of *Candida* colonization [7] and dental caries [8].

Many biochemical changes occur during pregnancy. For example, some studies have shown that salivary calcium depletion occurs in the third trimester, while the reduction of saliva phosphate starts from the second trimester. Decreasing the concentration of phosphate as a remineralizing agent affects the remineralization process. As a result, the salivary buffering capacity decreases during the first to the third trimester and increases the acidity of the oral environment [9].

Other studies have reported that a decrease in salivary pH in early pregnancy may be due to changes in the nutrition and oral hygiene of pregnant women [10–12]. Pregnant women often tend to use sweet snacks, but they do not change their daily oral hygiene habits. On the other hand, during pregnancy, due to hormonal changes and the consequent increase in gingival fluid flow, the susceptibility to periodontal disease and an increase in bleeding from the gums [13] makes mothers reluctant to follow daily oral hygiene such as brushing and flossing; Also, in the first weeks of pregnancy, due to changes in perception of smell and taste (especially the taste of toothpaste), nausea and boredom, maintaining good oral hygiene becomes difficult for a pregnant woman [14].

The results of various studies have mentioned different risk factors for dental caries among pregnant women, with dynamic and complex relationships between these factors [15–17]. In conclusion, it can be stated that there are controversial results on the relationship between the Decayed, Missing, and Filled Teeth (DMFT) index and pregnancy. Therefore, this issue is still a hypothesis requiring more comprehensive research. According to the dose–response principle proposed by Hill, if a correlation is observed between the number of pregnancies and the DMFT index, a justification for causal inference can be provided. Consequently, the present study intended to investigate the relationship between the number of pregnancies and the DMFT index obtained from the Tabari Cohort Study database. The Tabari Cohort Study (TCS), named after Tabaristan, the former name of Mazandaran province, was designed as part of the Prospective Epidemiological Research Studies in Iran (PERSIAN cohort study) for a better understanding of the risk factors associated with DMFT and the number of pregnancies.

## Methods

The present study was a cross-sectional, descriptive-analytical study conducted using the Tabari cohort study (TCS) data. The TCS sample was selected from the residents in the urban and the surrounding mountainous areas of Sari, Mazandaran Province, Iran. The data used were extracted from the enrollment phase of the TCS, which was a part of the Prospective Epidemiological Research Studies in Iran (PERSIAN) [18, 19]. The objectives, data collection tools, data collection methods, sampling method, and variables of the TCS. and PERSIAN were explained in the cohort profile and methodology studies [19, 20]. Moreover, at the beginning of the enrollment phase of TCS, the assessors attended training workshops for interview standardization and data collection methods. The TCS examines data related to adults; thus, the age range studied in this center includes people from 35 to 70 years old.

The TCS included 10,255 participants with an age range of 35–70 years, among which 6,106 female cases were entered into the present study. The inclusion criteria of our study were the data of all women participating in the TCS with a history of pregnancy, while the exclusion criteria were the data of women lacking a history of pregnancy or affected by malignancies. The women with malignancies were excluded due to their negligible number. Eventually, data for 5,496 eligible women were entered into the analysis using the census method.

This study was approved by the Ethics Committee of the Mazandaran University of Medical Sciences, Mazandaran, Iran. The data on the variables of age, age at the first pregnancy, body mass index (BMI), number of pregnancies, the total number of teeth at the time of examination, number of DMFT, socioeconomic status, educational level, employment status, and residence location, as well as the status of being affected by chronic and systemic diseases, including diabetes mellitus, hypertension, and cardiovascular diseases, were extracted in an Excel file to enter the data analysis of the present study. In addition, the DMFT index was calculated at the time of TCS implementation by oral examinations. A higher score in the DMFT index indicates more inappropriate dental health. Anthropometric measurements (height and weight) were also performed at the time of TCS implementation using the standard and calibrated instruments. Also, two trained nurses performed the oral examination of all subjects under the supervision of a quality control supervisor in the TCS clinic of Mazandaran University of Medical Sciences.

The socioeconomic status was determined using the principal component analysis (PCA). All Tabari cohort participants were classified into five groups based on 13 parameters, including area of residence (city or village), internal or foreign travels, activities such as reading books, having access to a computer and the internet, having a bathroom in their house, owning a car or motorcycle, and having some household appliances like a dishwasher, washing machine, freezer, colored television, and a vacuum cleaner. The total number of socioeconomic statuses was divided into five categories. Level 1 indicates the lowest level, level 2 is low, level 3 is average, level 4 is good, and level 5 represents the wealthiest level.

Being affected by diabetes mellitus was considered as a history of diabetes or a fasting blood sugar of ≥ 126 mg/dL in the laboratory tests. Being affected by hypertension was considered as a history of hypertension, a diastolic blood pressure of ≥ 90 mmHg, or a systolic blood pressure of ≥ 140 mmHg. Moreover, participants with a history of cardiovascular diseases were considered cardiovascular patients. It should be noted that the T.C.S. assessment team was provided with the medical records and the medical insurance booklet of the participants at the time of T.C.S. interviews.

Eventually, data were analyzed in the STATA software (version 14). The variables were described using the descriptive statistical indices of percentage (%), mean (M), Standard Deviation (S.D.), minimum (Min), maximum (Max), and quartile (Q), while the comparisons between the classified variables and the DMFT index were performed using the Pearson's correlation coefficient (univariate test) and the Analysis of Variance (ANOVA). Subsequently, the correlation between the number of pregnancies and the DMFT index was investigated using Pearson's correlation coefficient (univariate test) and the multivariate partial correlation test, which was used to adjust the effect of age. The DMFT index was considered as the quantitative dependent variable. Additionally, univariate linear regression analysis was used to investigate the effect of the number of pregnancies on the DMFT index without adjusting the effect of confounders, while multivariate linear regression analysis investigated this effect by adjusting the effect of confounders.

## Results

In the present study, among the total of 5,496 eligible participants, 68% (n = 3739) of the cases were urban residents, and 17.4% (n = 954) of them were employed. In terms of age, 15.9%, 35.4%, 31%, and 17.7% of the participants belonged in the age groups of 35–39, 40–49, 50–59, and 60–70 years, respectively. Given the socioeconomic status, 21.8% of the subjects belonged to socioeconomic Level 1, while socioeconomic Level 5 included 18.2% of the participants. It was also found that 43.7%, 40.3%, and 15.9% of the participants had a BMI value of ≥ 30, 25–29, and < 25, respectively. In terms of chronic diseases, the prevalence rates of diabetes, hypertension, and cardiovascular diseases were 18.9%, 25.7%, and 9.4%, respectively.

The DMFT index of the first quartile was estimated at the value of < 10. The statistical indices describing the number of pregnancies were calculated at 3.89 ± 2.19 and 1 and 18 for Min and Max, respectively. Furthermore, the fourth quartile of the number of pregnancies was obtained at the value of > 5.

The correlation between the number of pregnancies and the DMFT index was obtained at 0.48 using the Pearson's correlation coefficient (P < 0.001), while it was re-calculated to be 0.21 after adjusting the effect of age using the partial correlation test (*P* < 0.001).

It was concluded that the DMFT index was significantly higher in diabetic (19.84 ± 9.36 vs. 16.56 ± 8.89, *P* < 0.001) and hypertensive (19.80 ± 9.32 vs. 16.27 ± 8.80, P < 0.001) women than in non-diabetic and normotensive women, respectively. It was also significantly higher in the women suffering from cardiovascular diseases than those without cardiovascular diseases (21.23 ± 9.24 vs. 16.76 ± 8.95, P = 0.001) (Table 1).

**Table 1:** Relationship between the study variables and DMFT index

Variables	Sample number	Mean ± SD	*P *value
*Pregnancy number*			
One	351	12.74 ± 7.11	< 0.001*
Two	1327	13.09 ± 7.06	
Three	1252	14.80 ± 7.81	
Four	896	17.07 ± 8.11	
Five	568	19.82 ± 9.02	
six	410	22.89 ± 8.98	
More than six	692	26.17 ± 8.01	
*Age group (years)*			
35–39	875	10.62 ± 5.59	< 0.001*
40–49	1943	14.39 ± 7.29	
50–59	1705	19.22 ± 8.81	
60–70	973	25.09 ± 8.40	
*Hypertension*			
No	4083	16.27 ± 8.80	< 0.001*
Yes	1413	19.80 ± 9.32	
*Cardio-vascular disease*			
No	4978	16.76 ± 8.95	0.001*
Yes	518	21.23 ± 9.24	
*Diabetes mellitus*			
No	4460	16.56 ± 8.89	< 0.001*
Yes	1036	19.84 ± 9.36	
*Place of residence*			
Urban	3739	14.65 ± 7.82	< 0.001*
Rural	1757	22.56 ± 9.20	
*Has job*			
No	4542	17.70 ± 9.20	< 0.001*
Yes	954	14.69 ± 7.94	
*Educational status*			
University/college	907	12.29 ± 5.61	< 0.001*
9–12 years in school	1406	12.94 ± 6.47	
6–8 years in school	567	15.24 ± 7.95	
1–5 years in school	1508	18.46 ± 9.06	
No schooling	1108	25.81 ± 8.02	
*B.M.I*			
< 25	876	19.07 ± 9.87	< 0.001*
25–29.9	2217	17.22 ± 9.11	
≥ 30	2403	16.45 ± 8.61	
*Socio-economic status*			
1	1200	22.86 ± 9.30	< 0.001*
2	1117	18.70 ± 9.47	
3	1113	16.48 ± 8.56	
4	1067	13.59 ± 7.23	
5	999	13.27 ± 6.33	

*Significant

On the other hand, the DMFT index was significantly higher in women living in rural areas than in those in the urban areas (22.56 ± 9.20 vs. 14.65 ± 7.82), in unemployed women than in employed ones (17.70 ± 9.20 vs. 14.69 ± 7.94), and in those with low educational levels than in those with academic education (P < 0.001). Moreover, the DMFT index significantly decreased with improved socioeconomic status in those with socioeconomic levels 1 and 5, respectively (22.86 ± 9.30 and 13.27 ± 6.33. P < 0.001). It was also concluded that the DMFT values decreased significantly in women as the individual BMI increased (Table 1).

Eventually, the effects of potential confounding variables were adjusted, including age, age at the time of first pregnancy, BMI, residence location, socioeconomic status, employment status, educational level, and being affected by diabetes, hypertension, and cardiovascular diseases. Afterward, using the multivariate linear regression, it was shown that the DMFT index increased significantly by 34% for each unit increase in the number of pregnancies (Table 2). It also significantly increased by 33% for each year increase in the women's age. The results of univariate and multivariate linear regressions for the study variables are presented in Table 2.

**Table 2:** Relationship between the independent variables and DMFT index

	Univariate linear regression		Multiple linear regression	
	Beta	*P* value	Beta	*P* value
Age (continuous)	0.53	< 0.001*	0.33	< 0.001*
First pregnancy age (continuous)	− 0.33	< 0.001*	− 0.009	0.682
Pregnancy number (continuous)	2.00	< 0.001*	0.34	< 0.001*
BMI (continuous)	− 1.12	< 0.001*	− 0.17	< 0.001*
Has a job (References: no)	− 3.01	< 0.001*	− 0.34	0.185
Rural (references: urban)	7.90	< 0.001*	3.08	< 0.001*
*Educational status (references: university/college)*				
9–12 years in school	0.65	0.045*	− 0.32	0.309
6–8 years in school	2.95	< 0.001*	1.08	0.010*
1–5 years in school	6.16	< 0.001*	2.05	< 0.001*
No schooling	13.51	< 0.001*	4.19	< 0.001*
*Socio-economic status (references: level 1)*				
Level 2	− 4.16	< 0.001*	− 0.30	0.314
Level 3	− 6.38	< 0.001*	− 0.13	0.694
Level 4	− 9.26	< 0.001*	− 1.24	0.001*
Level 5	− 9.58	< 0.001*	− 1.45	< 0.001*
Diabetes mellitus (references: no)	3.27	< 0.001*	− 0.09	0.694
Cardio-vascular disease (references: no)	4.46	< 0.001*	− 0.11	0.722
Hypertension (references: no)	3.52	< 0.001*	− 0.098	0.674

*Significant

## Discussion

The present study intended to investigate the relationship between the number of pregnancies and the DMFT index among TCS participants.

In terms of educational level, Ibrahim El-Mahdi et al. reported a significant relationship between the educational level and oral health awareness among women. Moreover, they found that increased educational level could increase oral health awareness, although no significant relationship was observed between the educational level and oral hygiene performance [21]. These findings were consistent with those of our study. However, Haji Kazemi et al. found a significant relationship between oral health awareness and oral hygiene performance was [22].

In terms of socioeconomic status, Basgiru et al. reported poor socioeconomic status as a risk factor for dental caries in pregnant women [23], which was in line with the results of this research. This can be explained by the fact that women with poor socioeconomic status usually have more pregnancies, inappropriate diets, and poor oral hygiene. However, Shamsi et al. and Vergnes et al. reported that the relationship between the socioeconomic status and DMFT index was not significant [15, 16], which did not agree with our findings.

Based on the results of a study conducted by Shaghaghian et al., there was a positive correlation between the age and DMFT index showed in pregnant women, which was consistent with our results. This can be due to the accumulation of multiple risk factors over time. Moreover, they observed that the DMFT index was higher in pregnant women with multiple pregnancies before the current one [24]. These findings were also in line with those of studies performed by Allameh et al. [17], Radnai et al. [25], and Russel et al. [26].

In terms of chronic systemic diseases, such a diabetes mellitus, cardiovascular diseases, and hypertension, Azfar et al. reported that hypertension and gestational diabetes were positively correlated with dental caries in pregnant women in which gestational diabetes was found to cause enamel defects in newborns of diabetic women [27], which was inconsistent with our results indicating the lack of a significant relationship between DMFT index and these diseases.

Since cardiovascular diseases are among the most common causes of neonatal death during pregnancy and the presence of valvular heart disease increases the risk of pulmonary edema, women of childbearing age with cardiovascular disease should be constantly monitored. These women are encouraged to consult their doctor before becoming pregnant [28]. Studies have also shown that cardiovascular disorders can reduce the oral health-related quality of life (OHRQoL), especially in women; therefore, it seems that pregnant women should pay more attention to this issue than others [29].

Moreover, Surdacka et al. reported a significant positive correlation between the number of caries, plaque formation, and glycated hemoglobin (HBA1C) [30], which was compatible with findings of a study carried out by Azfar et al. However, Vieira et al. found no significant relationship between the HBA1C levels and the prevalence of caries in pregnant women [31]. On the other hand, they reported that having previous children was positively associated with the number of caries during pregnancy and postpartum. This can be explained by the fact that a higher number of children affect dental caries not only due to biological factors but also due to socioeconomic and behavioral factors since mothers of multiple children spend less time caring for themselves [26].

According to a meta-analysis conducted in 2017, the prevalence of depression after pregnancy was reported to be 11.9% [32]. It has been shown that anxiety, stress, and depression can directly affect neuro-endocrinal and immune system mechanisms and lead to an increased oral health risk by stimulating high-risk habits such as a cariogenic diet, reducing the frequency of brushing, etc. [33]. Therefore, the relationship between psychological status and the oral health of pregnant mothers is considered important. Increased salivary cortisol levels are also directly related to the amount of biofilm formed during pregnancy [34] and can lead to the formation of pathogen-induced plaque [35]. Therefore, a three-way relationship between stress, oral immune system, and microbial activity has been proposed, which is indicated by a decreased number of immunoglobulins and salivary flow and an increased microbial activity [36].

In another study, Vahdati et al. showed the mean DMFT index values of 5.9, 7.98, 8.5, and 8.25 in the women with 1, 2, 3, and 4 pregnancies, respectively, which was significant. It was revealed that women in their first pregnancy had lower DMFT than those with multiple pregnancies before, while the mean D.M.F. was similar in women with 3 and 4 pregnancies [37]. This difference can be explained by several factors, such as genetics, diet, daily oral hygiene, age, and general health. Moreover, studies have shown that, occasionally, dentists are reluctant to treat pregnant women due to fear of hurting the fetus, legal problems, or patient concerns [38].

## Limitations

One of the limitations of the present study was the fact that the data regarding the oral hygiene performance, including the history of brushing, flossing, using mouthwash since childhood, and accessing to and using dental care during and before pregnancy, were not available, while these variables were effective on DMFT as well. Also, the analysis did not include some subject-related variables, including oral health habits, fluoride intake, and diet habits. However, such variables as educational level, socioeconomic status, employment status, and residence location could reflect oral health indirectly and to some extent. Therefore, these variables may relatively solve the mentioned limitations. The lack of adequate facilities and dental staff can lead to underreporting of decayed teeth, but due to the fact that this restriction is not specific to any group and can be generalized to all the population of the study, it does not interfere with determining the relationship between the number of pregnancies and the DMFT index. The main concern about diagnosing decayed teeth is that the examiner may underreport the incidence of decayed teeth due to the lack of some specialized facilities and equipment such as radiography. However, it is noteworthy that this limitation exists in all of the samples (mothers with one, two, or three or more children). Although it is proposed as a limitation it will not affect the final relationship and conclusion of the study as it equally affects all participants of the present study.

## Recommendations

A large percentage of women experience pregnancy during their lifetime, and some give birth to more than one child. Women may face major oral health issues during pregnancy which may endanger the health of the mother and fetus. Therefore, to decrease this risk, it is recommended to start the preventive interventions and screenings in the first or second trimester. These measures include increasing the saliva pH by using neutralizing mouthwashes, carbohydrate intake reduction, Streptococcus mutans reduction through foods containing xylitol, and observing and promoting oral hygiene. Moreover, untreated caries in pregnant women increase salivary bacteria, increasing the risk of transmission of cariogenic bacteria to the baby and early childhood caries development [39]. Therefore, all pregnant women should receive appropriate dental care and oral health instruction. Moreover, they should undergo comprehensive assessments of their health. Women need the support of dentists, midwives, and family members, especially husbands or partners; these women can have a higher level of care during pregnancy.

## Conclusion

The present study showed a significant increase in the DMFT index with an increased number of pregnancies. In this respect, despite the cross-sectional design of the present study, it can be concluded that there is a potential causal relationship between these variables based on the dose–response principle proposed by Bradford-Hill. Furthermore, the secondary results of the study indicated that DMFT was also affected by age, B.M.I., residence location, educational level, and socioeconomic status.

## Data Availability

The datasets used and/or analyzed during the current study are available from the corresponding author on reasonable request.

## Abbreviations

DMFT: Decayed, Missing, and Filled Teeth
DMFS: Decayed, Missing, and Filled Surfaces
T.C.S.: Tabari Cohort Study
PERSIAN: Prospective Epidemiological Research Studies in Iran
B.M.I.: Body Mass Index
W.H.O.: World Health Organization
